# Influence of Age and Sex on the Clinical Profile of Metabolic Syndrome in Diabetic Patients

**DOI:** 10.7759/cureus.88376

**Published:** 2025-07-20

**Authors:** Rakesh Uppara Kadiyala, Mohd Asif, Swathi Uppara, Manuj Kumar Sarkar, Saphal Lakshmi Pasupulati, Nangadda Narmada, Sai Sujith Varma Datla, Rajeswari Murugesan, Suresh Vaikkakara, Raviteja Guddeti

**Affiliations:** 1 General Medicine, All India Institute of Medical Sciences, Mangalagiri, Mangalagiri, IND; 2 Psychiatry, All India Institute of Medical Sciences, Mangalagiri, Mangalagiri, IND; 3 General Medicine, All India Institute of Medical Sciences, Deoghar, Deoghar, IND; 4 Endocrinology, Diabetes and Metabolism, Narayana Health, Bangalore, IND; 5 Biostatistics, All India Institute of Medical Sciences, Mangalagiri, Mangalagiri, IND; 6 Endocrinology and Diabetes, All India Institute of Medical Sciences, Mangalagiri, Mangalagiri, IND; 7 Cardiovascular Medicine, Creighton University School of Medicine, Omaha, USA

**Keywords:** cardiovascular disease, diabetes mellitus, dyslipidemia, hypertension, lifestyle modifications, metabolic syndrome, obesity

## Abstract

Background and aim

In the 21st century, the rise in metabolic syndrome (MetS) prevalence has made it a global concern due to a higher risk of coronary artery disease and overall mortality, especially in India. Diabetic patients are more susceptible to metabolic syndrome. Therefore, it is crucial to determine the various risk factors associated with diabetes that contribute to the development of MetS. This study aimed to determine the prevalence of MetS and assess the risk factors and individual components of MetS in diabetic patients across both sexes and various age groups.

Material and methods

This cross-sectional prospective study was conducted in diabetic adults who visited the general medicine outpatient department at All India Institute of Medical Sciences (AIIMS), Mangalagiri. Patients who met the inclusion criteria were enrolled in this study. The collected data were analyzed according to the guidelines of the International Diabetes Federation (IDF) and the American Heart Association/National Heart, Lung, and Blood Institute (AHA/NHLBI) Joint Scientific Statement (2009) for MetS diagnosis.

Results

A total of 388 patients with diabetes mellitus were included in this study. The mean age was 52.55±11.82 years, with 20-59 years accounting for 69.6% (n=270) and ≥60 years for 30.4% (n=118). The proportion of males was 52.3% (n=203) of the study population. A high prevalence of MetS was reported (n=316; 81.4%). The prevalence of MetS was slightly higher in the 20-59 years age group (82.6%, n=223) compared to those ≥60 years (78.8%, n=93). A strong correlation between female sex and central obesity (n=168, 90.8%; p<0.001) and lower HDL-C levels (n=146, 78.9%; p<0.001) was present. A significant correlation was found between comorbidities, including hypertension (n=164, 86.3%, p=0.016), dyslipidemia (n=92, 88.5%, p=0.031), and the prevalence of MetS. Multivariable logistic regression analysis revealed that waist circumference (odds ratio {OR}: 1.1; p=0.016), systolic blood pressure (OR: 1.09; p=0.016), fasting blood glucose (OR: 1.02; p=0.044), and triglycerides (OR: 1.02; p=0.025) were independent predictors of MetS.

Conclusion

This study shows a high prevalence of metabolic syndrome (MetS) among diabetic patients, with females showing higher prevalence of MetS and its components, particularly central obesity and low HDL-C levels. The study also found statistically significant associations between MetS and glycemic parameters such as postprandial blood sugar and hemoglobin A1c (HbA1c). These findings emphasize the need for targeted screening and early intervention, particularly among female and younger diabetic patients. Public health initiatives focusing on lifestyle modifications and glycemic control are essential to mitigate MetS complications. Due to sociocultural diversities, region-specific studies are needed to develop localized guidelines for the effective management and prevention of metabolic syndrome.

## Introduction

Metabolic syndrome is considered an epidemic of the 21st century, constituting an interrelated group of metabolic abnormalities, including central obesity, insulin resistance, dyslipidemia, and hypertension [1,2]. MetS poses a global concern because of its high worldwide prevalence and strong association with the risk of developing diabetes mellitus. MetS has the potential to double cardiovascular disease risk and increase mortality [3,4]. In India, a recent rise in MetS can be attributed to lifestyle changes in both urban and rural areas compared to other South Asian countries [5]. Thus, early detection of MetS and understanding its risk factors are important for problem-specific interventions. Although the criteria for diagnosing MetS differ slightly, the definitions of MetS according to various criteria are discussed further. In certain populations, it has been found that the International Diabetes Federation (IDF) definition overestimates the prevalence of MetS compared to the National Cholesterol Education Program's Adult Treatment Panel III (NCEP ATP III), revised in 2005 by the American Heart Association (AHA) and the National Heart, Lung, and Blood Institute (NHLBI) definition, while the WHO definition underestimates it [6].

MetS is a multifactorial condition influenced by lifestyle, genetic, and environmental factors. Smoking and alcohol consumption are important lifestyle factors associated with MetS, with one potentiating the other [7]. Stress also has a major contribution through its direct effect on glucocorticoids, which in turn lead to insulin resistance and central obesity [8]. Thus, there is variation among populations due to differences in age, sex, ethnicity, lifestyle factors, and comorbidities [9]. Therefore, it is of utmost importance to identify the most prevalent risk factors associated with MetS. For example, studies conducted in Kerala and Assam have reported variability in the prevalence of MetS associated with lifestyle factors across different regions and populations in India [10,11]. The prevalence of MetS in Kerala was determined to be 24% in the general population, with 20% in males and 28% in females [11]. Similarly, a study in Dibrugarh, Assam, found a high prevalence with strong differences between sex and age groups [10]. A study conducted by Yadav et al. in central India found that the prevalence of MetS in diabetic patients was 45.8%, with prevalence rates of 41% and 54.1% in males and females, respectively [12]. Insights from the regional perspective are needed to plan necessary public health interventions.

There are a limited number of comparative studies in medical literature determining the trend of MetS in diabetic patients, especially in India, in relation to their demographic details and lifestyle factors [12]. In view of this, our study attempted to compare the prevalence, risk factors, and individual components of MetS in diabetic patients of different age groups and sexes. This study lays the groundwork for developing local health guidelines and strategies to mitigate the burden of Mets on public health.

## Materials and methods

Study design, setting, and population

This hospital-based, cross-sectional, prospective study was conducted in the General Medicine Outpatient Department (OPD) of the All India Institute of Medical Sciences (AIIMS), Mangalagiri, Andhra Pradesh. Patients with diabetes mellitus aged 18 years or older who gave consent were included in this study. Patients were excluded if they denied consent to participate, were pregnant, had hypothyroidism, or were taking steroids.

Sample size

The sample size was calculated as 385, with an expected prevalence (P) of 50%, a 95% confidence interval (CI), and a precision (d) of 5%. The formula used to calculate sample size is provided below.

$n = \frac{Z^2 \cdot P(1 - P)}{d^2}$

Here, n is the sample size, and Z is the statistic corresponding to the level of confidence.

Data collection

Following approval by the Institutional Ethics Committee, All India Institute of Medical Sciences, Mangalagiri, patients with diabetes who met the specified inclusion and exclusion criteria were included in this study. Informed written consent was obtained from the participants and data were collected in a semi-structured proforma regarding the patient’s demographic details, comorbidities and past medical history (diabetes, hypertension, dyslipidemia, chronic obstructive pulmonary disease {COPD}, coronary artery disease {CAD}, cerebrovascular accident {CVA}), treatment history, personal history (smoking, alcohol, exercise, sleep, diet, menstrual history), anthropometric measurements (height, weight, BMI, waist circumference), vital data (temperature, pulse rate, respiratory rate, blood pressure), and examination findings. Waist circumference was measured in a horizontal plane around the abdomen at the level of the highest point of the iliac crest, at the end of a normal expiration with a standard measuring tape to the nearest 0.1 cm [13]. The formula of weight (kilograms) divided by the square of the person’s height (meters) was used to calculate the body mass index (BMI). Blood pressure was measured manually using a calibrated mercury sphygmomanometer with a standard-sized cuff on the right arm of participants. Blood investigations, including fasting blood sugar, postprandial blood sugar, HbA1c, and lipid profile, were done in the study population.

Diagnostic Criteria for Metabolic Syndrome

The metabolic syndrome was defined in accordance with the criteria established by the Joint Interim Statement of the International Diabetes Federation Task Force on Epidemiology and Prevention; National Heart, Lung, and Blood Institute; American Heart Association; World Heart Federation; International Atherosclerosis Society; and International Association for the Study of Obesity in 2009 [13].

At least three of the following components are required for the diagnosis of MetS: (1) abdominal obesity (waist circumference ≥90 cm for men or ≥80 cm for women). (2) Triglyceride levels ≥150 mg/dL or drug treatment for elevated triglycerides. (3) HDL cholesterol ≤40 mg/dL for men or ≤ 50 mg/dL for women or on drug treatment for reduced HDL. (4) Systolic/diastolic blood pressure ≥130/85 mmHg or receiving hypertension-specific drug treatment. (5) Fasting plasma glucose ≥100 mg/dL or on drug treatment for elevated glucose. The definitions of MetS according to various criteria are given in Table 1 [13,14].

**Table 1:** Various criteria for diagnosis of MetS. WHO: World Health Organization; NCEP: National Cholesterol Education Program; IDF: International Diabetes Federation; WC: waist circumference; FPG: fasting plasma glucose; DM: diabetes mellitus; M: male; F: female; SBP: systolic blood pressure; DBP: diastolic blood pressure; IGT: impaired glucose tolerance; IFG: impaired fasting glucose; WHR: waist to hip circumference ratio; BMI: body mass index; MetS: metabolic syndrome

Component	WHO (1999)	NCEP (2001)	IDF (2005)	Harmonized criteria (2009)
Central obesity	Optional: WHR >0.90 (M), >0.85 (F) or BMI >30 kg/m² or WC elevated	WC ≥102 cm (M), ≥88 cm (F)	Mandatory: WC ≥90 cm (M), ≥80 cm (F) for South Asians (ethnic-specific)	WC ≥90 cm (M), ≥80 cm (F) for South Asians (ethnic-specific)
Insulin resistance/glucose	Mandatory: IGT, IFG, DM, or insulin resistance	FPG ≥100 mg/dL or diagnosed DM or on its treatment	FPG ≥100 mg/dL or diagnosed DM or on its treatment	FPG ≥100 mg/dL or diagnosed DM or on treatment
Triglycerides	≥150 mg/dL	≥150 mg/dL or on its treatment	≥150 mg/dL or on its treatment	≥150 mg/dL or on treatment
HDL cholesterol	HDL <35 mg/dL (M), <39 mg/dL (F)	<40 mg/dL (M), <50 mg/dL (F) or on its treatment	<40 mg/dL (M), <50 mg/dL (F) or on its treatment	<40 mg/dL (M), <50 mg/dL (F) or on treatment
Blood pressure	≥140/90 mmHg or on treatment	SBP ≥130 or DBP ≥85 mmHg or on its treatment	SBP ≥130 or DBP ≥85 mmHg or on antihypertensive treatment	SBP ≥130 or DBP ≥85 mmHg or on treatment
Microalbuminuria	Urinary albumin ≥20 μg/min or albumin-creatinine ratio >30 mg/g	-	-	-
Diagnostic threshold	Insulin resistance + any 2 of the remaining risk factors	Any 3 of 5 criteria	Central obesity + any 2 of 4 remaining factors	Any 3 of 5 criteria

Statistical analysis

Data were interpreted using descriptive analysis with IBM SPSS Statistics version 24.0 (Armonk, NY: IBM Corp.) for Windows. In the statistical analysis of demographic details, frequency, proportions, and the Pearson chi-square test were used. While analysing the anthropometric measurements and biochemical investigations, mean with SD and independent Student's t-test/Mann-Whitney U test were used. A p-value of <0.05 is considered the level of significance. Logistic regression analysis was used to compare groups and assess associations between significant predictors and MetS.

## Results

Sociodemographic characteristics

The current study included 388 patients with diabetes mellitus (DM) aged ≥18 years. The mean age was 52.55±11.82 years. The age groups were categorized as follows: 20-59 years (69.6%, n=270) and ≥60 years (30.4%, n=118). In this study, the number of males was greater than that of females: 203 (52.3%) vs. 185 (47.7%), which is representative of the general population. A significantly high prevalence of MetS was found in 81.4% of the study population (n=316). Trends of MetS across different sociodemographic profiles, comorbidities, lifestyles, and family history are shown in Table 2.

**Table 2:** Comparison of sociodemographic parameters, past medical, personal, and family histories in diabetic patients with and without MetS. *P<0.05 is statistically significant. MetS: metabolic syndrome

Characteristics	Metabolic syndrome absent	Metabolic syndrome present	Chi-square test statistic	p-Value
Age (mean: 55±11.82) years				
20-59 years	47 (17.4%)	223 (82.6%)	0.776	0.378
≥60 years	25 (21.2%)	93 (78.8%)		
Sex				
Male	51 (25.1%)	152 (74.9%)	12.147	<0.001*
Female	21 (11.4%)	164 (88.6%)		
Medical history				
Hypertension				
No	46 (23.2%)	152 (76.8%)	5.849	0.016*
Yes	26 (13.7%)	164 (86.3%)		
Dyslipidemia				
No	60 (21.1%)	224 (78.9%)	4.631	0.031*
Yes	12 (11.5%)	92 (88.5%)		
Coronary artery disease				
No	66 (18.6%)	288 (81.4%)	0.020	0.866
Yes	6 (17.6%)	28 (82.4%)		
Cerebrovascular accident				
No	69 (18.5%)	303 (81.5%)	0.000	0.984
Yes	3 (18.8%)	13 (81.2%)		
Thyroid disorder				
No	61 (19.1%)	259 (80.9%)	0.309	0.578
Yes	11 (16.2%)	57 (83.8%)		
Personal history				
Smoking				
Non-smoker	44 (16.5%)	222 (83.5%)	3.321	0.19
Ex-smoker	11 (28.2%)	28 (71.8%)		
Current smoker	17 (20.5%)	66 (79.5%)		
Alcohol				
Non-alcoholic	49 (16.6%)	246 (83.4%)	3.086	0.079
Alcoholic	23 (24.7%)	70 (75.3%)		
Sleep				
Inadequate for age	49 (18.5%)	216 (81.5%)	0.002	0.961
Adequate for age	23 (18.7%)	100 (81.3%)		
Exercise				
No exercise	45 (19.3%)	188 (80.7%)	1.087	0.581
<30 mins/day	16 (15.4%)	88 (84.6%)		
≥30 mins/day	11 (21.6%)	40 (78.4%)		
Diet				
Vegetarian	12 (22.2%)	42 (77.8%)	0.558	0.455
Mixed diet	60 (18.0%)	274 (82.0%)		
Yes	2 (6.7%)	28 (93.3%)		

Age and metabolic syndrome

Prevalence of MetS was slightly higher in the 20-59 years age group (82.6%, n=223) when compared to ≥60 years group (78.8%, n=93). Components of MetS were not significantly different across the age groups between 20-59 and ≥60 years, except hypertriglyceridemia (n=105, 38.9%) vs. (n=165, 61.1%), which had a statistically significant difference (p=0.008). Central obesity was observed in 81% (20-59 years: n=221, ≥60 years: n=96) of patients in both age groups. The findings have been summarized in Table 3.

**Table 3:** Age and components of metabolic syndrome. *P<0.05 is statistically significant. HDL-C: high-density lipoprotein cholesterol; BP: blood pressure; FBS: fasting blood sugar

Parameters	20-59 years (n=270)	≥60 years (n=118)	Chi-square test statistic	p-Value
Abdominal obesity				
Absent	49 (18.1%)	22 (18.6%)	0.014	0.907
Present	221 (81.9%)	96 (81.4%)		
Triglycerides				
<150 mg/dL	105 (38.9%)	63 (53.4%)	7.033	0.008*
≥150 mg/dL	165 (61.1%)	55 (46.6%)		
Low HDL-C				
Yes	178 (65.9%)	67 (56.8%)	2.952	0.086
No	92 (34.1%)	51 (43.2%)		
BP				
<130/85 mmHg	137 (50.7%)	68 (58.6%)	1.563	0.211
≥130/85 mmHg	133 (49.3%)	50 (42.4%)		
FBS				
<100 mg/dL	19 (7%)	10 (8.5%)	0.245	0.620
≥100 mg/dL	251 (93%)	108 (91.5%)		

**Figure 1:**
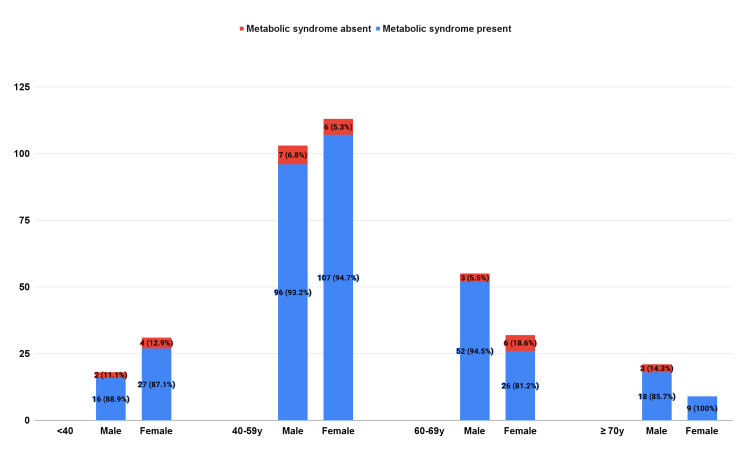
Trends of metabolic syndrome in both sexes and various age groups.

The prevalence of MetS was stratified based on the age groups (years): <40, 40-59, 60-69, and ≥70 years (Figure 1). The prevalence of MetS in females aged 40-59 years was significantly higher than that in those aged 60-69 years (n=104, 94.7% vs. n=26, 81.2%, p=0.038).

Sex and metabolic syndrome

Females had a higher prevalence of MetS when compared to males (n=164, 88.6% vs. n=152, 74.9%; p<0.001), which was statistically significant. Thus, a strong correlation was established between sex and MetS. The correlation between sex and components of MetS is summarized in Table 4.

**Table 4:** Sex and components of metabolic syndrome. *P<0.05 is statistically significant. HDL-C: high-density lipoprotein cholesterol; BP: blood pressure; FBS: fasting blood sugar

Parameter	Males	Females	Chi-square test statistic	p-Value
Abdominal obesity				
Absent	54 (26.6%)	17 (9.2%)	19.628	<0.001*
Present	149 (73.4%)	168 (90.8%)		
Triglycerides				
<150 mg/dL	96 (47.3%)	72 (38.9%)	2.763	0.096
≥150 mg/dL	107 (52.7%)	113 (61.1%)		
Low HDL-C				
No	104 (51.2%)	39 (21.1%)	37.808	<0.001*
Yes	99 (48.8%)	146 (78.9%)		
BP				
<130/85 mmHg	109 (53.7%)	96 (51.9%)	0.126	0.722
≥130/85 mmHg	94 (46.3%)	89 (48.1%)		
FBS				
<100 mg/dL	16 (7.9%)	13 (7.0%)	0.102	0.749
≥100 mg/dL	187 (92.1%)	172 (93.0%)		

Abdominal obesity was noted in 90.8% (n=168) of females and 73.4% (n=149) of male diabetic patients, with a p<0.001, which was statistically significant. Lower HDL levels were noted in 78.9% (n=146) females and 48.8% (n=99) males, with p<0.001, which was statistically significant.

Comorbidities and lifestyle

**Figure 2:**
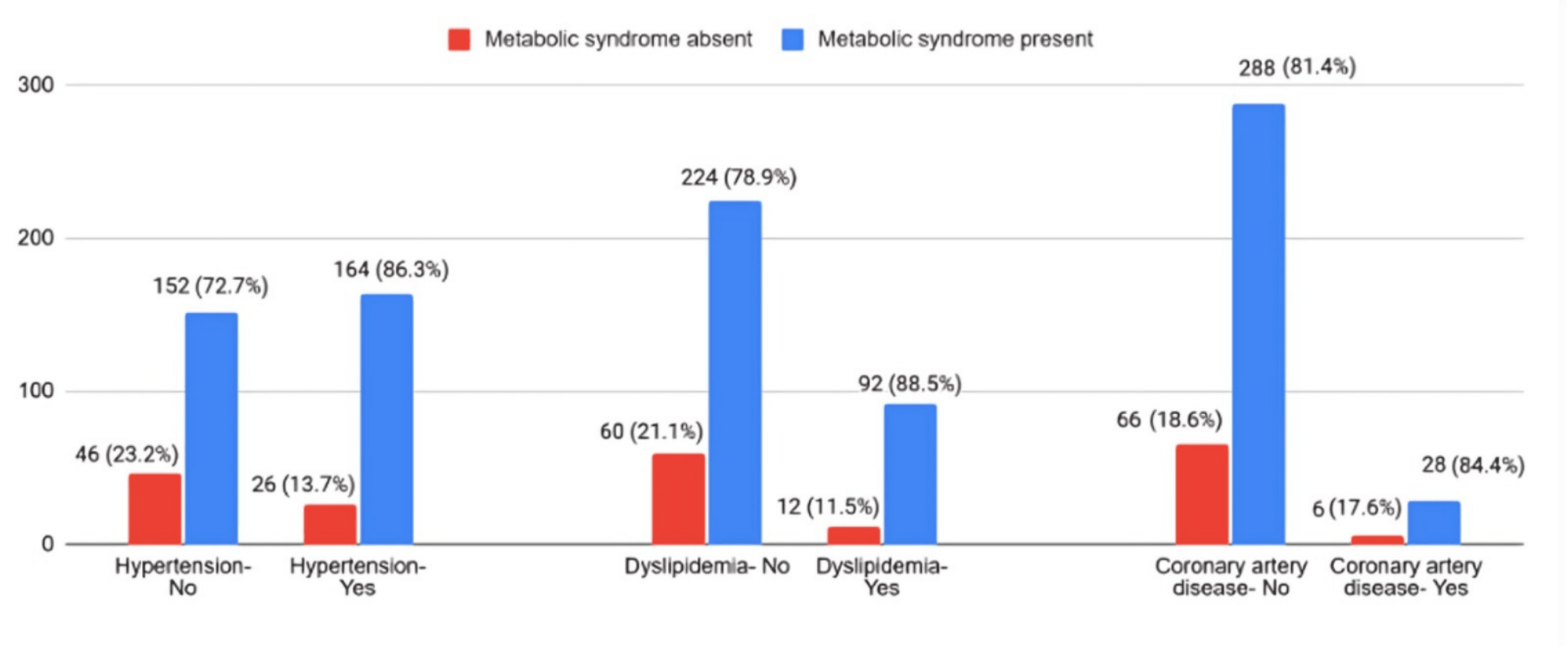
Metabolic syndrome and comorbidities.

A history of hypertension was found in 86.3% (n=164) of patients with MetS, which was higher than in patients without a history of hypertension (n=152, 76.8%; p=0.016), which was a statistically significant difference. A history of dyslipidemia was found in 88.5% (n=92) of patients with MetS, which was higher than in patients without dyslipidemia (n=224, 78.9%; p=0.031), a statistically significant difference. The correlation between MetS and other comorbidities, such as cerebrovascular accident (p=0.984) and hypothyroidism (p=0.578), was statistically insignificant. Figure 2 illustrates the significant comorbidities associated with MetS. No significant correlation was found between family history of coronary artery disease, diabetes, or hypertension and MetS.

**Figure 3:**
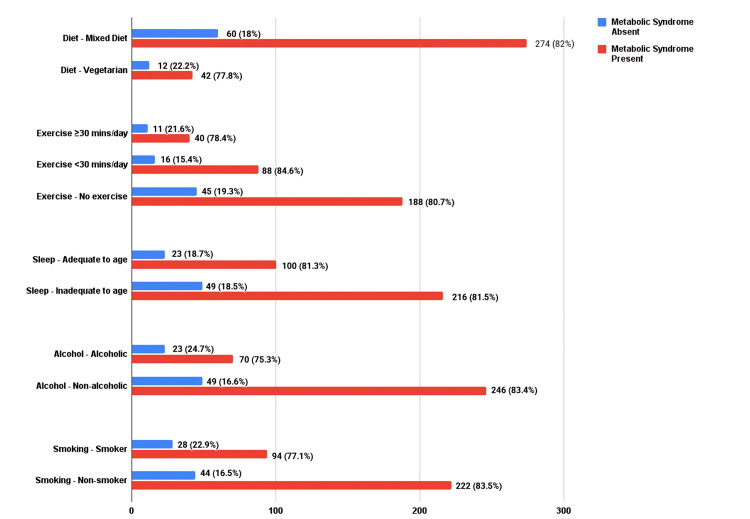
Comparison of lifestyle factors in patients with and without MetS. MetS: metabolic syndrome

Smoking and alcohol consumption were found in 21.4% (n=83) and 24% (n=93) of the study population, respectively. A large proportion of the study population lacked any sort of dedicated exercise (n=233; 60.1%). In addition, inadequate sleep duration according to age was noted in 68.3% (n=265). Notably, 76% (n=295) of the population was obese, with a body mass index ≥30 kg/m^2^. Abdominal obesity, a salient feature of MetS, was more prevalent in patients with diabetes (n=317; 81.7%). Dietary patterns were predominantly mixed (86.1%, n=334), with a smaller proportion (13.9%, n=54) adhering to a vegetarian regimen. MetS was slightly lower in vegetarians than in diabetics consuming a mixed diet, but the correlation was statistically insignificant. In diabetic patients, 19.1% (n=74) had regular menstrual cycles, and 25.5% (n=99) had attained menopause. Comparison of lifestyle factors in patients with and without MetS is shown in Figure 3.

Biochemical profile and BMI in metabolic syndrome

**Figure 4:**
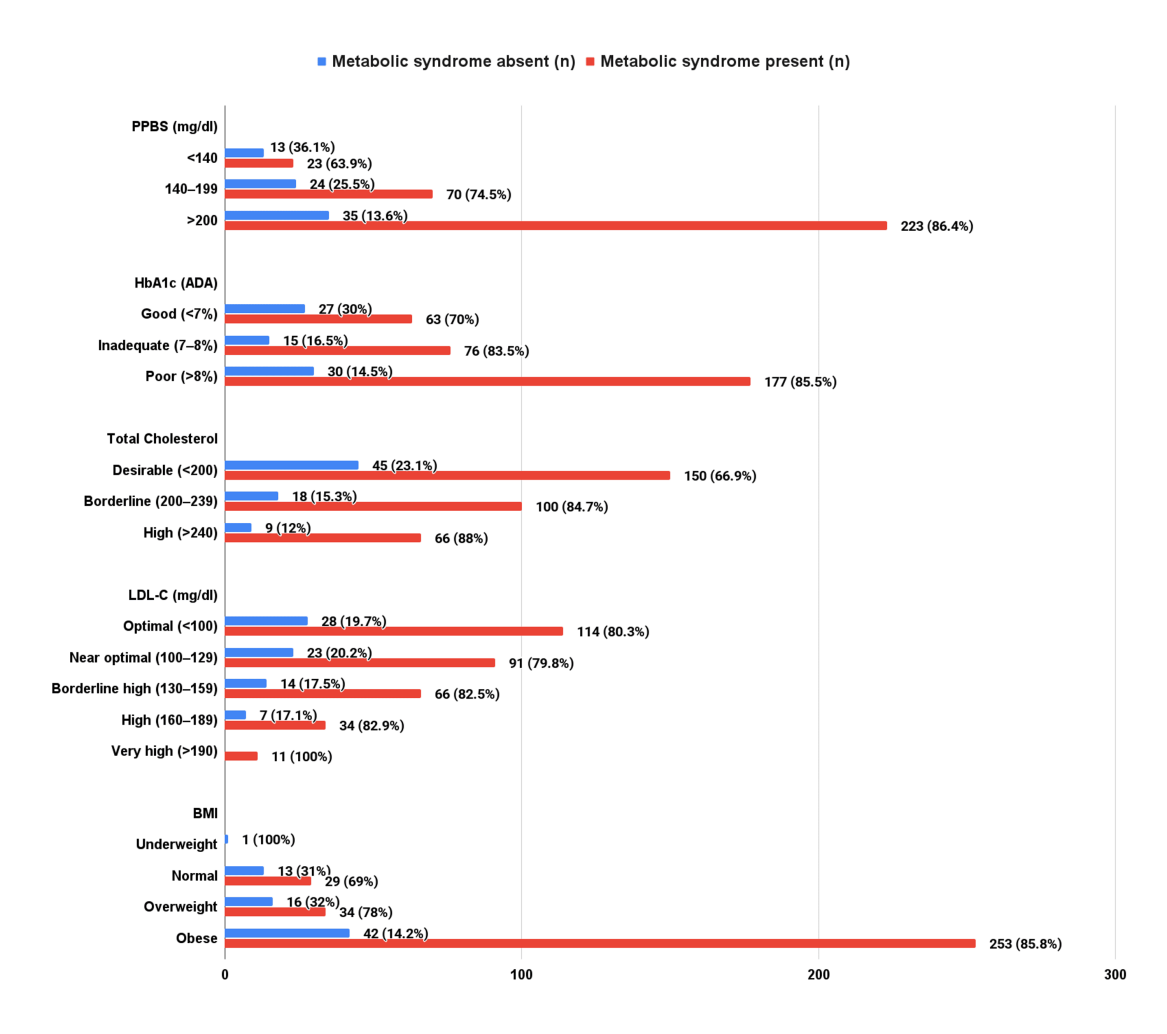
Biochemical profile and BMI in metabolic syndrome. P<0.05 is statistically significant. PPBS: postprandial blood sugar; LDL-C: low-density lipoprotein cholesterol; BMI: body mass index

A total of 56.7% (n=220) of patients with diabetes had high triglyceride levels ≥150 mg/dL, and 63.1%(n=245) had low HDL-C levels. Half of the participants had abnormal total cholesterol levels (n=193; 49.7%). Elevated fasting blood glucose levels were present in 92.5% (n=359), and poor glycemic control with higher HbA1c levels was observed in 53.4% (n=207) of the study population. Trends of metabolic syndrome indicated by various biochemical tests and BMI are illustrated in Figure 4.

A total of 70.5% (n=223) of the MetS patients exhibited higher postprandial blood sugar (PPBS) levels ≥200 mg/dL compared to PPBS levels <140 mg/dL (n=23, 7.3%), which is statistically significant (p=0.001). In the study population, only 30.2% (n=90) had HbA1c ≤7, and 69.8% (n=298) had HbA1c >7, and the correlation between MetS and HbA1c was statistically significant (p=0.006).

Individuals without MetS were more likely to exhibit desirable rather than elevated total cholesterol levels (n=45, 62.5% vs. n=9, 12.5%; p=0.06). In the non-MetS group, 38.9% (n=28) achieved optimal low-density lipoprotein cholesterol (LDL-C) levels, a finding that was also observed in the MetS group (n=114; 36.1%). Notably, none of the individuals without MetS had high LDL levels, whereas 11 individuals (3.5%) with MetS did. However, this difference was not statistically significant.

About 10.8% (n=42) of participants had normal BMI, while 76% (n=295) of participants fell into the category of obesity. Abdominal obesity, as measured by waist circumference, was observed in a substantial proportion of participants (81.7%, n=317). The majority of individuals with MetS were overweight or obese rather than having a normal BMI (n=287, 90.8% vs. n=29, 9.2%; p<0.001), which was statistically significant.

Predictors of metabolic syndrome

**Figure 5:**
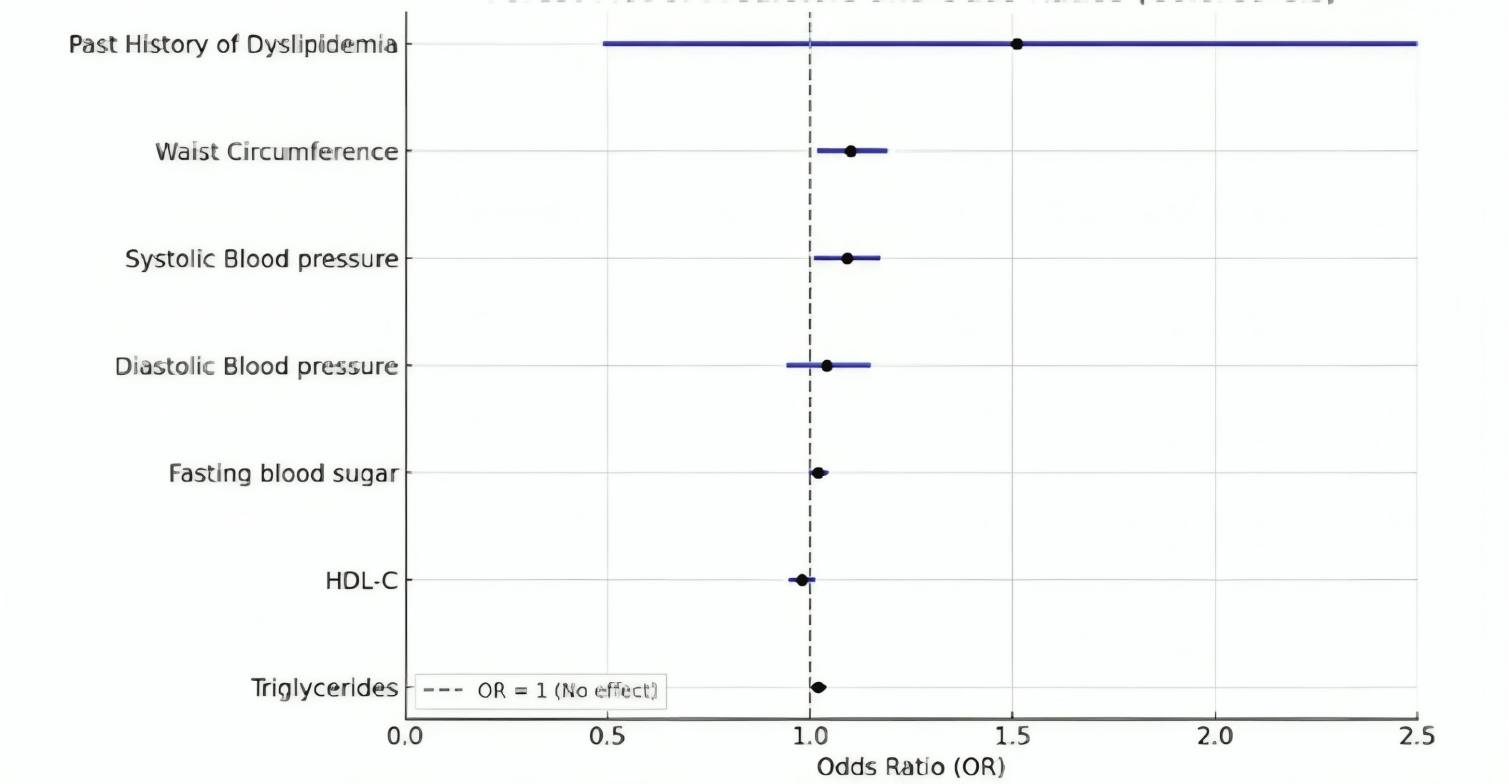
Forest plot depicting the possible predictors of metabolic syndrome. HDL-C: high-density lipoprotein cholesterol

Multivariate logistic regression analysis was performed across the determined predictors of metabolic syndrome (Figure 5). In summary, the statistically significant independent predictors of MetS were waist circumference (odds ratio {OR}: 1.1; p=0.016), systolic blood pressure (OR: 1.09; p=0.016), fasting blood glucose (OR: 1.02; p=0.044), and triglyceride levels (OR: 1.02; p=0.025).

Frequency of components in metabolic syndrome

**Figure 6:**
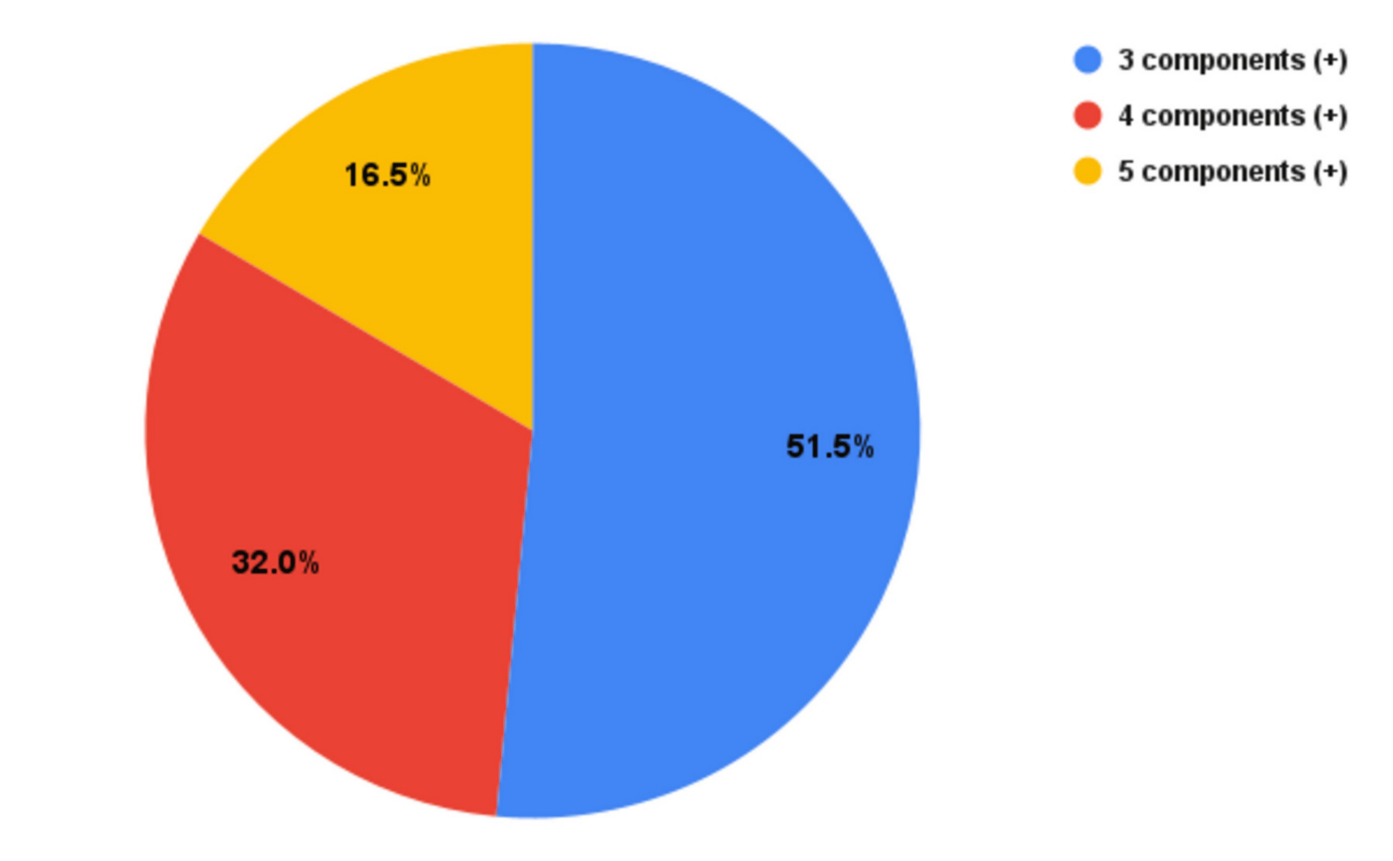
Number of components associated with MetS criteria. MetS: metabolic syndrome

The number of components present to fulfill the MetS criteria is shown in Figure 6. Over half (n=200) of them had three components, 32% (n=124) had four components, and 16.5% (n=64) had all five components.

## Discussion

Metabolic syndrome (MetS) with cardiometabolic risk factors, such as central obesity, dyslipidemia, hypertension, and hyperglycemia, has emerged as a major public health concern in India. It has been established that MetS is significantly more prevalent in individuals with diabetes. Its presence exacerbates insulin resistance and increases the risk of coronary artery disease and overall mortality [14].

In this cross-sectional study involving 388 individuals with diabetes mellitus, the observed prevalence of MetS was 81.4% (n=361). Surana et al. conducted a study involving 5088 patients from urban Mumbai, which yielded a prevalence of 77.2% (n=3928) [15]. Yadav et al. noted a prevalence of 45.8% among 700 patients in central India [12]. In a study conducted among 410 North Indians from Haryana, the prevalence of metabolic syndrome varied from 76.1% to 26.3% according to the IDF, Harmonized, NCEP-ATP III, and WHO criteria, respectively [16]. The precise prevalence of metabolic syndrome in the South Indian population with diabetes mellitus was lacking, which was found to be 81.4% in our study.

Trends from different parts of the world yielded results showing a lower prevalence of MetS in patients with diabetes mellitus, on average, than that found in our study. In a study conducted by Pokharel et al. among 1061 Nepalese patients with diabetes mellitus, the prevalence of MetS was reported to be 80.3%, 73.9%, 69.9%, and 66.8% according to the Harmonized, NCEP-ATP III, WHO, and IDF definitions, respectively [17]. Asghar et al. reported a prevalence of 65% for MetS in 160 Pakistani individuals with diabetes [18]. Alawdi et al. reported that 87.5% of type 2 diabetic patients in Yemen had metabolic syndrome [19]. African meta-analysis has shown that the pooled prevalence of metabolic syndrome (MetS) in diabetes mellitus was 53% in Ethiopia and 63.1% in sub-Saharan Africa (NCEP-ATP III) and 60.8% in sub-Saharan Africa (IDF criteria) [20,21].

In this study, sex emerged as a strong determinant of MetS in diabetes mellitus, with a significantly higher prevalence among females (88.6%) compared to males (74.9%). This is in line with various studies showing a higher prevalence of MetS with age and in females [17,18]. Bhattarai et al. showed a prevalence of metabolic syndrome ranging from 91% to 95% in females compared to males (71-82%) according to various criteria [22]. In Ethiopia, the prevalence of MetS was 48% in females and 32% in males. A systematic review by Musilanga et al. also revealed a higher prevalence in females than in males (72-73% vs. 44.5-50%) [21]. In Alawdi et al.'s study, an increased prevalence of metabolic syndrome was observed with age (71.2% in patients under 35 years vs. 96% in patients above 75 years) and in females (91.3%) [19].

In this study, central obesity was more prevalent in females (90.8% vs. 73.4%). Furthermore, females exhibited higher incidences of elevated triglyceride levels (61% vs. 52.7%), low HDL cholesterol levels (78.9% vs. 48.8%), elevated fasting blood glucose levels (93% vs. 92%), and elevated blood pressure (48% vs. 46.3%) compared to males. The study by Alawdi et al. demonstrated a higher prevalence of abdominal obesity in females than in males (84% vs. 42.6%) and lower HDL levels, predominantly in females (97.3% vs. 92.3%) [19]. Our findings are consistent with these results. Surana et al. reported an increased prevalence of central obesity (80%) and hypertension (77.6%) in females, with males showing a higher prevalence of hypertension (88.4%) and hypertriglyceridemia (74%), although their findings on hypertension and hypertriglyceridemia were conflicting [15]. Bhattarai et al. observed trends of abdominal obesity (95% vs. 77%), hypertension (90% vs. 72%), low HDL levels (81% vs. 56%), and elevated triglyceride levels (4% vs. 61%) in females compared to males, with their findings on triglycerides also conflicting with our study [22]. These observed sex differences in abdominal obesity, lipid profiles, and blood pressure underscore the significance of sex-specific factors in the development of MetS.

The prevalence of MetS was slightly higher in the 20-59 years age group (82.6%) compared to those ≥60 years (78.8%). In the components of MetS, elevated serum triglyceride levels (≥150 mg/dL) were present in 61.1% of individuals aged 20-59 years, compared to 46.6% in those aged ≥60 years. In the 20-59 year age group, 65.8% of individuals exhibited low HDL-C levels, compared to 56.8% in those aged 60 years and above. Elevated blood pressure was present in 49.3% of individuals, and elevated fasting blood glucose levels were observed in 93% of individuals aged 20-59 years. The prevalence of each component of metabolic syndrome (MetS) in the diabetic population aligns with findings from other similar studies on MetS [20,21]. Generally, the prevalence of hypertension and abnormal lipid profiles increases with age in diabetic individuals [23,24]. However, our study's findings align with those of Al-Mukhtar et al., indicating that the younger diabetic population (<60 years) exhibited a higher prevalence of MetS [25].

This study showed a strong correlation between MetS and glycemic parameters like postprandial blood sugars and HbA1c, indicating that measures to manage these parameters may be a key strategy in the prevention and management of MetS. Additionally, comorbidities such as dyslipidemia and hypertension were found to be significantly associated with MetS, highlighting the syndromic nature of MetS and its association with various cardiovascular risk factors. While a lack of physical activity was evident in individuals with MetS, no significant correlation was observed between alcohol consumption and smoking.

In this study, three components of MetS were present in 51.5%, four components in 32%, and all five components of MetS were present in 16.5% of the patients with metabolic syndrome. Surana et al. showed that 19% of patients with metabolic syndrome had all five components of metabolic syndrome, 36.35% had four components, and 44.6% had three components, which is in line with our findings [15]. The majority of Nepalese patients with diabetes mellitus had a cluster of four components of metabolic syndrome, including central obesity, high triglyceride levels, low serum HDL, and hypertension [17]. Asghar et al. noted a combination of three components in 43.4%, four components in 36.2%, and five components in 20.4%, findings similar to those in our study [18]. In the study by Alawdi et al., three components were identified in 24% of patients, four components in 33.8% of patients, and five components in 43.4% of patients [19]. This underscores the growing burden of metabolic abnormalities in our population, possibly due to shifting dietary patterns, sedentary lifestyles, and increasing rates of obesity.

The high prevalence of MetS among those with cardiovascular comorbidities like coronary artery disease and stroke, although not statistically significant, aligns with existing literature that positions MetS as a precursor to adverse cardiovascular outcomes. This emphasizes the need for early identification and management of MetS components to reduce long-term morbidity. In summary, this study highlights a very high prevalence of MetS in a semi-urban South Indian population with diabetes mellitus. In this study, significant associations were found between MetS and female sex, obesity, glycemic control, hypertension, and dyslipidemia.

Limitations

The study population consisted of patients with diabetes who attended AIIMS, Mangalagiri. Therefore, the findings may not be broadly applicable to the general population due to sociocultural diversity.

## Conclusions

In conclusion, this study highlights the notably high prevalence of metabolic syndrome (MetS) among diabetic patients. The findings underscore the significant influence of sex, with females exhibiting a higher prevalence of MetS and its components, particularly central obesity and low HDL-C levels. The study also found statistically significant associations between MetS and glycemic parameters such as postprandial blood sugars and HbA1c.

These findings emphasize the need for targeted screening and early intervention strategies, particularly among female and younger patients with diabetes, to address the growing burden of MetS and its associated cardiovascular risks. Public health initiatives focusing on lifestyle modification, stress management, and improved glycemic and lipid control are essential to mitigate the long-term complications of MetS in the diabetic population. Due to sociocultural diversities, region-specific studies are warranted to develop localized guidelines and policies for effective management and prevention of metabolic syndrome.
